# Injury Prevention Considerations for Drum Kit Performance

**DOI:** 10.3389/fpsyg.2022.883279

**Published:** 2022-05-10

**Authors:** Nadia R. Azar

**Affiliations:** Department of Kinesiology, Drummer Mechanics and Ergonomics Research (DRUMMER) Laboratory, University of Windsor, Windsor, ON, Canada

**Keywords:** drummer, drum kit, playing-related musculoskeletal disorders, risk factors, injury prevention, education, performance

## Abstract

For any skilled performer to deliver their optimal performance, preparation must extend beyond task-specific skill development to include psychological skills training, physical conditioning, and injury prevention. The keynote lecture upon which this article is based (delivered at the International Symposium on Performance Science 2021) explored current research that demonstrates the importance of physical conditioning and injury prevention for drummers (i.e., percussionists who play the drum kit). Early results revealed that professional drummers’ heart rates during live performances can reach similar levels to those of other professional athletes during competitions. They also established that playing-related musculoskeletal disorders (PRMDs) are very common in drummers, particularly those affecting the upper limbs such as tendinitis and carpal tunnel syndrome. Evidence from laboratory-based studies supports non-neutral postures, repetitive movements, and exposure to hand-arm vibration as risk factors for the development of these injuries in drummers. Embedding injury prevention education within drum kit curricula is a promising strategy for reducing the rates at which drummers report experiencing PRMDs, and the barriers and facilitators that drum kit educators encounter when attempting to do so are currently under investigation. When drummers include both physical conditioning and injury prevention within their overall preparation regimen, they will maximize their potential to deliver their peak performance.

## Introduction

Becoming an elite performer of any task or activity takes years of intensive preparation. The preparation required to achieve this level of performance includes four domains. *Task-specific skill development* is where performers spend the bulk of their preparation time, for obvious reasons: one must be highly skilled at the task to deliver an elite performance. *Psychological skills training* helps performers prepare to handle the psychological demands of the task, such as learning how to manage performance anxiety. *Physical conditioning* involves developing the physical capacity to perform a task through specialized training programs, such as strength or endurance training. Finally, *injury prevention* involves taking steps to identify and manage the risks of injury associated with a task. All these domains of preparation contribute to the ability to deliver an optimal or peak performance, but the keynote presentation upon which this article is based focused on the importance of physical conditioning and injury prevention for drummers (delivered at the International Symposium on Performance Science 2021: https://youtu.be/oFCrd1Pc9Qc).

## Physical Conditioning

Skilled drummers possess many performance-enhancing physical and cognitive attributes compared to non-drummers, such as greater movement speed (Fujii and Oda, 2006), lower levels of muscular co-contraction (Fujii et al., 2009), enhanced motor readiness and proactive inhibitory control (Bianco et al., 2017), and enhanced error detection (Petrini et al., 2011). Although no studies have compared strength, fitness, or endurance attributes between skilled drummers and non-drummers, the former most likely possess superior cardiovascular fitness and endurance, given the substantial physiological demands imposed upon them while drumming. Professional and semi-professional drummers expend approximately 500–600 kilocalories per hour (De La Rue et al., 2013; Brown, 2016; Romero et al., 2016; Azar, 2021a), which is considered “vigorous” physical activity [American College of Sports Medicine (ACSM) et al., 2018]. Based on heart rate (HR) data collected during live performances, De La Rue et al. (2013) suggested that drumming was an intermittent activity that relied on both the aerobic and anaerobic energy systems. Furthermore, my own data (Azar, 2021a) demonstrated that the average HR of professional drummers during live performances was comparable to that of professional soccer players during competitive match play when expressed as a percentage of maximum HR [79% (Azar, 2021a) vs. 86% (Torreño et al., 2016)].

These studies clearly showed that playing the drums is a high-intensity and intermittent activity, and that professional drummers are likely to exceed current recommended exercise targets for healthy adults [Garber et al., 2011; Canadian Society for Exercise Physiology (CSEP), 2020] while they are out on tour. Therefore, they should consider engaging in cardiovascular endurance and high-intensity interval training to prepare their bodies to handle the physical demands of their profession and to deliver their peak performance. Unfortunately, this is an area that is overlooked by many drummers (Azar, 2021b). This is problematic because physical conditioning is not only important for achieving peak performance, but also an important component of injury prevention.

## Injury Prevention

Effective injury prevention strategies can only be achieved when the mechanisms by which musculoskeletal disorders develop and the factors that lead to (or prevent) their development (i.e., risk factors) are understood. Musculoskeletal injuries occur when the demands placed on a tissue exceed its capacity to meet them (Price, 2021). The greater the demands, and the lower the capacity, the greater the risk that an injury will occur. Musculoskeletal injuries can manifest in two ways. Acute injuries occur when a single event loads a tissue beyond its capacity (Potvin, 2011; e.g., a slip on an icy sidewalk resulting in a wrist fracture). Chronic injuries are the result of repeated sub-maximal loading (Potvin, 2011) that wears down the tissue so that over time, its capacity to handle loading is reduced to the point where a load that was once acceptable is no longer tolerated and an injury occurs (e.g., stress fractures in long distance runners). Playing-related musculoskeletal disorders (PRMDs) fall into the latter category. They develop over time as a direct result of playing an instrument and interfere with a musician’s ability to play their instrument at the level to which they are accustomed (Zaza et al., 1998). Examples include tendinitis, carpal tunnel syndrome (CTS), bursitis, and low back pain (Whiting and Zernicke, 2008).

If injuries occur when demands exceed capacity (Price, 2021), then to reduce the risk of developing PRMDs, one must reduce the demands placed on the musculoskeletal tissues and increase the tissues’ capacity to meet those demands. Tissue capacity can be increased through engagement in physical conditioning, but this alone may not be enough to match demands to capacity and prevent an injury (Price, 2021). There are several possible approaches to matching demands to capacity, one of which involves identifying one’s exposures to PRMD risk factors and reducing them to levels that are within one’s capacity. Injury prevention initiatives are most likely to be successful when a combination of these (and other) approaches is implemented (Price, 2021). However, very little research has examined PRMDs and identified their risk factors in percussionists, and even less has focused on these issues for drummers, specifically. The following sub-sections describe the research my laboratory has undertaken to identify drummer-specific rates and patterns of PRMDs and the biomechanical risk factors that may lead to (or reduce the risk of) their development. These data will ultimately be used to develop prevention strategies to reduce the prevalence and burden of PRMDs in this population.

### Rates and Patterns of PRMDs in Drummers

The lifetime prevalence of PRMDs in percussionists (including drummers) was previously reported to be 74–77%, and the upper limbs and the low back tended to be affected the most (Sandell et al., 2009; Hawkins, 2015). However, the results of these and other studies are difficult to apply to drummers for a variety of reasons (e.g., study populations consisting of mixed percussion groups, small sample sizes, and lack of inclusion of diverse musical genres). Therefore, I designed a study to address these limitations (Azar, 2020), the goal of which was to determine the rates and injury patterns of PRMDs specifically in drummers and to identify characteristics that might put drummers at risk for (or protect them from) developing PRMDs.

The methods and results of this study are described in detail elsewhere (Azar, 2020). Briefly, I developed an electronic survey that included three types of questions. The first section asked about respondent characteristics (e.g., age, gender, and years of playing experience). The second section included a definition of PRMDs and asked participants about their experiences with PRMDs (e.g., injury history, pain levels, and how much the injury interfered with their ability to play). It also included a body map so participants could indicate which body parts were affected, and a question asking them to list the medical diagnosis they had received, if any. The definition I provided to the participants is as follows (adapted from Zaza et al., 1998):

*Playing-related musculoskeletal problems are defined as “pain, weakness, numbness, tingling, or other symptoms that interfere with your ability to play the drums at the level to which you are accustomed.” This definition does not include mild playing-related aches and pains that come and go, nor does it include pain unrelated to playing your instrument (e.g., pain from injuries sustained in a car accident, sports injuries, and slipping and falling on an icy sidewalk).*

The third section of the survey asked a series of questions about drumming- and lifestyle-related characteristics (e.g., practice and performance habits and how often they engaged in warm-ups/cool-downs and exercise).

The survey was distributed through social media and online for a period of 3 months, yielding 831 valid responses. 90% of the respondents self-identified as being male, and the mean age was 40 years (SD = 14 years). The data were analyzed using qualitative and quantitative approaches where appropriate, and the primary results were as follows:

Sixty-eight percent of the respondents reported a lifetime history of PRMD. This was lower than the rates reported previously in guitar players (Fjellman-Wiklund and Chesky, 2006: 81%) and non-percussion orchestral musicians (Berque et al., 2016: lifetime prevalence of 80% when percussionists were removed from the sample), but was consistent with the rates reported previously in brass instrumentalists (Chesky et al., 2002: 61%), and most relevantly, in drummers/percussionists (Hawkins, 2015: 77%; Sandell et al., 2009: 74%). The respondents also reported similar body regions where PRMDs developed, particularly the upper limbs and the back. Twenty-three percent of the respondents reported having experienced a PRMD within the 7 days prior to completing the survey. These respondents were also asked to rate how much the PRMD interfered with various aspects of their drumming and their daily lives, and the average rating was five out of 10 (i.e., a moderate level of interference). Considering the high stakes involved in a career as a musician (Sandell et al., 2009), a moderate degree of PRMD interference is concerning. Other alarming findings included the large proportion of respondents who reported a lifetime history of multiple PRMDs (59%) and that only 42% of the respondents with a history of PRMDs received diagnoses from a medical professional. The two most common were tendinitis and CTS.

This study confirmed that PRMDs are a significant health problem for drummers. This is compounded by a lack of extended healthcare benefits, as most survey respondents reported that they do not have these benefits (35%), or they get them through other employment (36%). This highlights the importance of determining the risk/protective factors for these injuries to reduce their burden. The data also clearly showed that the upper limbs were where most of the problems were occurring and that the most common injuries were tendinitis and CTS. This provided a roadmap for additional studies to examine the potential risk factors for PRMDs in more depth.

### Drummer-Specific Biomechanical Risk Factors for PRMDs

Biomechanical risk factors are the mechanical demands placed on the body that increase one’s risk of injury (e.g., forceful exertions, repetitive movements, non-neutral or static postures, and exposure to vibration). In the cases of tendinitis and CTS, both are associated with forceful exertions, repetitive movements, and non-neutral postures, especially when they occur in combination (Bernard, 1997). CTS has also been associated with exposure to hand-arm vibration (HAV; Bernard, 1997). Although several studies have examined the biomechanics and motor control of drumming (e.g., Fujii et al., 2009, 2011; Dahl, 2011; Chong et al., 2016; Mutio et al., 2017; Eriksen et al., 2018), only three have examined this data in the context of injury prevention. Salvalaio et al. (2011) used thermography to examine leg muscle activation in one drummer during two double-kick pedal techniques. Based on the thermographic profiles, the authors concluded that both techniques were likely to lead to muscular fatigue and repetitive stress injuries with prolonged use. Cuden et al. (2015) used self-reported anthropometric data from male Filipino drummers (ages 14–30 years) and information about the frequency of use of various drum kit elements to propose guidelines for drummers to set up their drum kits to promote more neutral upper limb postures while drumming. However, they based their study on the assumption that drummers assume non-neutral upper limb postures while playing and did not quantify drummers’ postures themselves. Roseiro et al. (2018) quantified hand-arm vibration (HAV) exposure in six drummers, and they reported HAV exposures at levels that could put drummers at increased risk of developing PRMDs. However, their study was limited by a small sample size and by a short data collection time (180 s), and they did not specify whether the drummers played an actual song or just a repeated pattern. Thus, these are two areas in which my research laboratory is working to fill the gap.

#### Non-neutral Postures and Repetitive Movements

Recently, we used three-dimensional motion capture technology to document the movement patterns of drummers’ upper limbs while playing (Flammia and Azar, 2021). Eleven drummers participated in this study, nine of whom yielded useable data. Once instrumented (Xsens MVN Awinda, Xsens Technologies B. V, Enschede Netherlands), participants played four songs in randomized order. Three songs were pre-determined by the research team for standardization, and one was chosen by the participants to represent their typical performance style. Joint angle-time histories were exported for the primary axes of rotation of the shoulder, elbow, and wrist joints (bilaterally). The average, minimum, and maximum joint angles, the total ranges of motion (ROM), and the percent of playing time spent in non-neutral joint postures were calculated for each standardized song individually and for all three songs combined.

**Figure 1:**
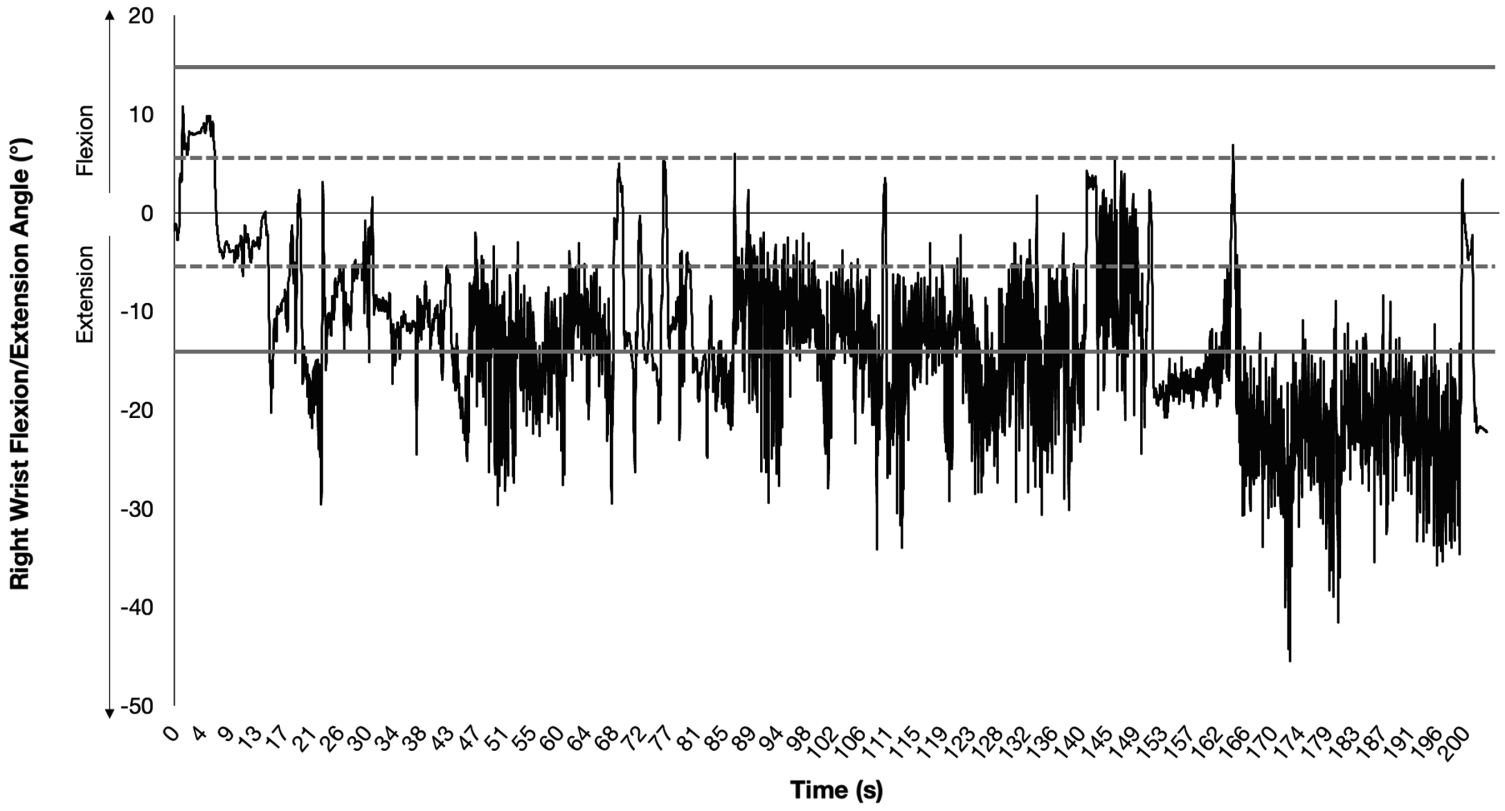
Flexion/extension posture of a drummer’s right wrist over the course of playing a single song (approximately 180 s duration). The horizontal axis represents time, and the vertical axis represents the angle of a drummer’s wrist flexion/extension posture. Flexion angles are positive and extension angles are negative. The area between the dotted lines is the range of wrist flexion/extension angles that were considered “neutral” wrist posture, and the areas between the dotted and thick solid lines is the range of wrist flexion/extension angles that were considered “mildly non-neutral” wrist posture. Participants spent a substantial amount of time with their wrists extended well beyond this range, clearly in the territory that would be considered non-neutral and even extreme.

One notable finding from this study was how much time the drummers spent not only with their wrists outside of neutral flexion/extension postures (mean: 90% for both hands), but with their wrists in extension (mean: 95% for the right hand and 96% for the left hand; see Figure 1). Similar patterns were observed for wrist radial/ulnar deviation and for some shoulder movements.

These data also highlighted the presence of repetitive movements, especially at the wrists. Each peak and valley on the graph in Figure 1 indicate a change in the direction of the motion of the wrist within a single, three-minute song. Thus, the data from this study suggest that non-neutral postures and repetitive movements are likely to be contributing factors for tendinitis and CTS in drummers, especially given that they occur in combination.

#### HAV Exposure

My laboratory is building on the study conducted by Roseiro et al. (2018) by monitoring HAV exposure in more drummers in more authentic playing conditions (Durward, in progress). The participants were instrumented with a triaxial integrated circuit piezoelectric accelerometer to record accelerations (m/s^2^) in the anteroposterior, mediolateral, and longitudinal directions at the hand (SEN041F, PCB Piezotronics Inc.; Provo, Utah, United States). They then played three songs of their choosing that represented their typical playing style. The data were extrapolated based on the participants’ self-reported daily playing time and compared to recognized industrial standards for hand-arm vibration exposure [American Conference of Governmental Industrial Hygienists (ACGIH), 2019].

Preliminary results from six participants revealed HAV exposures that exceeded the American Conference of Governmental Industrial Hygienists (ACGIH), (2019) action limit of 2.5 m/s^2^, and four participants’ exposures exceeded the threshold limit value (TLV) of 5.0 m/s^2^. If vibrations of these magnitudes were recorded during an industrial task, the company would have to remove the tool from the workplace until a new tool could be selected and/or the task could be redesigned to bring the exposure level to within the TLV (Wasserman, 2008). Although preliminary, the data from this study suggest that drummers’ exposures to HAV while playing are likely to play a role in the development of CTS in this group.

### Modifying the Risk of PRMDs

There are several steps that can be taken to reduce exposures to these risk factors, even if they cannot be eliminated completely. For example, it is not possible to eliminate repetitive motion from drumming, but it is possible to reduce the influence of the repetitive motions by introducing breaks during practices and taking microbreaks during the pauses between songs during performances. The best time to take these steps is before an injury develops, rather than trying to deal with them reactively. Thus, drummers should learn about injury prevention early in their drumming careers, and this is where the role of drum kit educators becomes apparent.

#### Injury Prevention Education

Embedding health promotion/injury prevention within post-secondary instrumental music programs can positively influence music students’ knowledge and attitudes toward health and engagement in healthy behaviors (Spahn et al., 2001; Barton and Feinberg, 2008; Zander et al., 2010; López and Martínez, 2013; Árnason et al., 2018; Matei et al., 2018) and reduce the frequency and severity of the symptoms of PRMDs (Spahn et al., 2001; López and Martínez, 2013; Davies, 2020). These studies were not specific to drum kit education, but a second analysis of the data from the PRMD survey suggested that this might also be the case for drummers.

The methods and results of this analysis are described in detail elsewhere (Azar, 2021b). Briefly, I separated the survey respondents into two groups based on whether they had received injury prevention education (PrevEd) during their formal drum training. I then compared the rates of reporting both lifetime and current PRMDs between the groups and calculated odds ratios to determine the magnitudes of the effects.

Eighty-one percent of the survey respondents reported having received formal training (FT) on the drums, but only 42% of those reported that they had received PrevEd. Within the “PrevEd” group (i.e., the group that reported having received PrevEd during FT), the proportion who reported a lifetime history of PRMD was significantly smaller than the proportion who reported no lifetime history of PRMD (*p* < 0.05). The opposite trend was observed in the “NoPrevEd” group (i.e., the group who reported they did not receive PrevEd or had not undertaken FT). Drummers who had not received PrevEd were approximately twice as likely to report a lifetime history of PRMDs than those who had (odds ratio = 1.97). The same trends were observed in respondents who reported they were currently experiencing a PRMD (odds ratio = 1.64). Drummers in the “PrevEd” group also reported engaging in warm-ups, cool-downs, and exercise significantly more often than those in the “NoPrevEd” group (*p* < 0.05).

However, the “NoPrevEd” group also included drummers who had received FT. These participants were likely trained in the use of proper drumming technique, posture, movement qualities, etc. Even if these were not specifically presented as techniques to reduce the risk of PRMDs, it is possible that this training could have had this effect. To account for this, I separated the “NoPrevEd” group into three sub-groups: “FT, no PrevEd received” (“FT-NoPrevEd”), “FT, unsure whether PrevEd received,” and “no FT/no response.” When I re-ran the analysis, I found that the trend held as: in the “FT-NoPrevEd” group, the proportion who reported a lifetime history of PRMD was significantly larger than the proportion who reported no lifetime history (*p* < 0.05). This trend also held for those reporting a current PRMD. These data suggest that FT alone might not be enough to reduce the rates of PRMDs in drummers and that educators must specifically address injury prevention to have a substantial impact on the rates at which drummers report PRMDs.

In addition to the quantitative analysis, I also conducted a qualitative analysis of the participants’ responses to a question asking them to describe what they were taught about injury prevention during their FT. The responses were grouped into various themes and categories, but many were too generic to yield much insight. For example, one participant simply stated “stretching.” However, the type (i.e., static or dynamic), timing (i.e., before or after playing), and duration of the stretching can influence its effects on both performance and injury prevention (Chaabene et al., 2019; Emery and Panasen, 2019). Drummers may not experience the full benefits of these activities if they are not engaging in their optimal forms. Further to this point, the respondents who reported receiving PrevEd were less likely to report a history of PRMD, but their rates of reporting PRMDs were still high, and even though they engaged in injury prevention behaviors more often than the other groups, they still did so infrequently. They reported that they warmed up before playing “about half the time,” they cooled down after playing “sometimes” or “never,” and they engaged in exercise “less than 1 h” per week. This suggests that the respondents might not have engaged in these behaviors often enough, or they might not have optimized how they engage in these behaviors, to reap their full benefits.

#### Barriers and Facilitators to Including PrevEd Within Drum Kit Curricula

PrevEd alone might not be the “magic bullet” for reducing the rates of PRMDs in drummers, but research suggests that it can have an impact beyond that of FT alone. The fact that so few of the survey respondents reported having received PrevEd from their instructors was both intriguing and concerning. The recommendations to include musician wellness and occupational health within music education have been around for at least two decades (Palac, 2008). Thus, there appears to be a gap between what could be considered “best practice” and what is occurring in actual practice. Therefore, the next step in my laboratory’s research plan is to identify the barriers and facilitators to include PrevEd within drum kit curricula. We will interview 30 drum kit educators about the reasons why they do or do not teach their students PrevEd, and what resources are available or are needed to help them to do so effectively. This work will guide the creation of resources and strategies that will empower these educators to develop or enhance this aspect of their curricula. Over time, as more educators begin including PrevEd in their curricula, we expect to see a reduction in rates of reporting PRMDs and in the burden of PRMDs from both economic and quality of life perspectives.

## Summary and Conclusion

Playing the drums is a physically demanding task, and physical conditioning to be able to handle the demands is essential. PRMDs are very common in drummers, particularly in the wrists and the low back. The two most frequently reported medical diagnoses for these injuries are tendinitis and CTS, and evidence that the known biomechanical risk factors for these two injuries are present in drumming at levels that could lead to their development is beginning to accumulate. PrevEd taught by drum kit educators shows promise as a PRMD prevention strategy, but more information about the reasons why these educators do or do not include it in their teaching, and what resources they might need to be able to do so effectively, is needed to maximize the potential of this strategy to reduce the burden of PRMDs in drummers.

Earlier, I described four domains of preparation to achieve elite performance: Task-specific skill development, psychological skills training, physical conditioning, and injury prevention. These last two domains are complementary, because physical conditioning is an important component of injury prevention. To reduce the risk of developing a PRMD, the demands placed on musculoskeletal tissues must be reduced and their capacity to handle those demands must be increased. Over time, engaging physical conditioning will increase tissues’ capacity to handle demands. At the same time, taking steps to identify and reduce exposures to risk factors for developing PRMDs will reduce the demands being placed on the tissues. When drummers include both physical conditioning and injury prevention within their overall preparation regimen, they will maximize their potential to deliver their peak performance.

## Data Availability Statement

The datasets presented in this article are not readily available because participants only gave consent to the use of their data within the author’s own research group. The data cannot be added to repositories or made available by request. Questions regarding the datasets should be directed to azar5@uwindsor.ca.

## Ethics Statement

The studies involving human participants were reviewed and cleared by the University of Windsor Research Ethics Board University of Windsor, Windsor, ON, Canada. The participants in the laboratory-based and field studies provided their written informed consent to participate in these studies. Survey respondents implied their consent when they completed and submitted their survey responses.

## Author Contributions

The author confirms being the sole contributor of this work and has approved it for publication.

## Funding

The work described herein was funded by the University of Windsor (Internal Start-up Grant, Research Grants for Women), from the proceeds of NA’s publications with The Drumeo Beat, and with in-kind support from Evans Drumheads. NA has also received funding from the GRAMMY Museum Foundation, Inc. (Scientific Research Grants) to conduct the future work described herein.

## Conflict of Interest

NA is a contributor to The Drumeo Beat, a free online blog for drummers. Her laboratory also receives in-kind support from Evans Drumheads.

## Publisher’s Note

All claims expressed in this article are solely those of the authors and do not necessarily represent those of their affiliated organizations, or those of the publisher, the editors and the reviewers. Any product that may be evaluated in this article, or claim that may be made by its manufacturer, is not guaranteed or endorsed by the publisher.
